# Endoscopic ultrasound-directed transenteric ERCP in patients with benign and malignant diseases and inaccessible biliary system

**DOI:** 10.1055/a-2858-0711

**Published:** 2026-05-19

**Authors:** Leonie Grossmann, Thomas Veiser, Philipp Euler, Nicole Balasus, Tobias Dertmann, Maximilian Schneider, Torsten Beyna

**Affiliations:** 1 Department of Internal Medicine and Gastroenterology 39751 Evangelisches Krankenhaus DüsseldorfDüsseldorfNRWGermany

**Keywords:** Pancreatobiliary (ERCP/PTCD), ERC topics, Endoscopic ultrasonography, Intervention EUS, Stones, Strictures

## Abstract

**Background and study aims:**

Patients requiring biliary interventions with endoscopically inaccessible papilla or biliodigestive anastomosis represent a challenge in clinical practice. Historically, percutaneous transhepatic biliary drainage (PTBD) has often been the only viable treatment after failed endoscopic attempts. Recently, endoscopic ultrasound (EUS)-guided techniques have emerged as promising alternatives. This study evaluated the feasibility and safety of EUS-guided transenteric ERCP (EDEE) at a tertiary referral center.

**Patients and methods:**

This retrospective, single-center study included patients who underwent EDEE between February 2020 and November 2025. Primary endpoints were safety and overall technical success, defined as successful creation of an EUS-guided anastomosis (EA) using a lumen-apposing metal stent (LAMS) followed by endoscopic retrograce cholangiopancreatography (ERCP) via LAMS. Secondary endpoints included clinical success, need for reinterventions, and procedure characteristics.

**Results:**

A total of 24 patients (70.3 ± 12.6 years; 6 female) were analyzed. Sixteen patients had surgically altered anatomy (5 Whipple, 11 Roux-en-Y), and eight presented with malignant gastric outlet obstruction. EA and ERCP via LAMS were conducted in two stages with a median interval of 29.5 days. In 15 patients, the afferent loop was accessed after filling via a previously placed PTBD, which was removed after EDEE. Technical success was 100% (24/24) for EA and 87.5% (21/24) for ERCP via LAMS; overall technical and clinical success were 87.5% (21/24). Procedure-related complications occurred in two patients (8.3%), including one aspiration pneumonia and one LAMS misplacement.

**Conclusions:**

This study suggests that EDEE is a feasible and safe method for biliary drainage in patients with inaccessible biliary anatomy, demonstrating high technical and clinical success in both malignant and benign conditions being performed at a highly specialized tertiary center.

## Introduction

Endoscopic retrograde cholangiopancreatography (ERCP) remains the gold standard and first-line approach for biliary interventions, such as endoscopic diagnosis and treatment of biliary strictures 1 or treatment of biliary stone disease 2 . However, in certain clinical scenarios – such as presence of malignant or benign gastric outlet obstruction (GOO) or surgically altered anatomy – standard ERCP becomes technically unfeasible due to endoscopic inaccessibility of the papilla or biliodigestive anastomosis (BDA) 3 4 . Common reconstructive techniques after Whipple’s pancreaticoduodenectomy or bilioenteric anastomoses typically include Billroth II gastrojejunostomy and Roux-en-Y anastomoses. Although ERCP may still be feasible in a subset of patients with Billroth II anatomy using standard side-viewing duodenoscopes, most cases involving Roux-en-Y reconstructions preclude standard endoscopic access and interventions.

In such situations, alternative techniques and strategies have been introduced, which are generally considered more complex and invasive, including device-assisted enteroscopy (DAE) 5 6 7 , percutaneous biliary drainage 8 , and laparoscopic-assisted ERCP 9 . Technical and clinical success rates and complication risks vary depending on technique, endoscopist experience, and individual patient-related factors 10 . As a further limitation, not every intervention can be successfully performed with each of these techniques, particularly if recurrent access and interventions are needed. Percutaneous biliary drainage (PTBD), for example, is associated with repeated interventions over extended periods and may, in some instances, result in permanent external drainage 11 12 .

In recent years, endoscopic ultrasound (EUS)-guided techniques, such as EUS-guided hepaticogastrostomy (EUS-HGA) or EUS-guided choledochoduodenostomy (EUS-CDS) have emerged as promising alternatives for biliary access in this patient population 13 14 15 16 . Although these procedures demonstrate high technical success rates of approximately 90%, their application is limited in complex situations, such as presence of multiple strictures at the level of the BDA or pathologies involving the right biliary system 17 18 19 20 21 .

More recently, EUS-assisted gastrointestinal anastomoses using lumen-apposing metal stents (LAMS) have been introduced to create an endoscopic bypass in cases of malignant or benign gastrointestinal obstruction 22 . Furthermore, an EUS-directed technique has been used to facilitate regular ERCP with standard duodenoscopes after Roux-en-Y gastric bypass (RYGB) creating transgastric access to the excluded stomach (EUS-directed transgastric ERCP [EDGE]) with excellent outcomes 23 . Although creation of a gastro-gastrostomy between the pouch and the remnant, excluded stomach during EDGE has been shown to be safe and effective, data from EUS-directed transenteric ERCP (EDEE) in patients with postsurgical anatomy other than RYGB are limited 24 25 26 .

This study aimed to assess clinical and technical outcomes of EDEE using LAMS in a real-world setting for managing biliary diseases in patients with surgically altered anatomy other than RYGB or with inaccessible papilla due to (malignant) GOO.

## Patients and methods

### Study design

This retrospective, single-center study was conducted at a German endoscopic tertiary referral center with extensive experience in endoscopic management of biliopancreatic diseases using ERCP, PTBD-, EUS- and enteroscopy-assisted techniques. Data were collected from February 2020 to November 2025. The study protocol was approved by the Institutional Review Board (Ethikkommission Ärztekammer Nordrhein, Germany, reference 155/2025) and was registered in the U.S. National Library of Medicine database (clinicaltrials.gov, identifier: NCT07096895).

### Study objectives and outcome measures

Primary objectives were overall technical success rate (number and rate of patients with successful creation of an EUS-guided anastomosis (EA) (gastroenteric or enteroenteric) followed by a successful ERCP including all planned interventions) and safety (number and rate of patients in whom a procedure-related complication occurred).

Secondary endpoints included the clinical success rate, defined as absence of need for alternative drainage – indicating that ERCP performed via LAMS provided adequate biochemical improvement and symptom resolution. In addition, need for reinterventions and technical aspects of both procedures – the EA and the ERCP performed via LAMS – also were evaluated as secondary endpoints.

All patients who required endoscopic biliary intervention and in whom the papilla or the BDA could not be reached by standard ERCP or device-assisted-enteroscopy-assisted ERCP (DAE-ERCP) due to endoscopic failure and, therefore, underwent EDEE at the study site during the study period, were included. Patients with surgically altered anatomy who were not eligible for standard ERCP and had an indication for biliary intervention that could not be performed through an enteroscopic approach at that time (e.g., cholangioscopy, electrohydraulic lithotripsy) and, therefore, underwent EDEE primarily were also eligible for inclusion.

Exclusion criteria were successful biliary intervention achieved using an (endoscopic) approach other than EDEE. Due to the retrospective design of the study, no further exclusion criteria were defined.

### Data management and statistical analysis

All patients who fulfilled the inclusion criteria were retrospectively registered and enrolled. The database was created using Microsoft Excel (Microsoft Corporation, Redmond, Washington, United States). Data entry was performed by trained study nurses in the Department of Gastroenterology, Evangelisches Krankenhaus Düsseldorf and verified by a physician. Statistical analyses were carried out using SAS version 9.3 or higher (SAS Institute Inc., Cary, North Carolina, United States). Continuous measures were summarized with sample size, mean, median, standard deviation, minimum, and maximum. Categorical measures were presented with the counts and percentages of subjects in each category.

### Study interventions

EDEE was performed following a standardized technical protocol while accounting for patient individual anatomical configuration, ultimate aim of the intervention, and need for reinterventions as well as underlying pathology under discretion of the endoscopist. The protocol included four steps 1) preparation and access to the target jejunal loop, the following steps; 2) EUS-guided creation of the gastro-/enteroenteric anastomosis using free-handed technique and LAMS deployment; 3) removal of access devices; and 4) ERCP via LAMS.

**Fig. 1:**
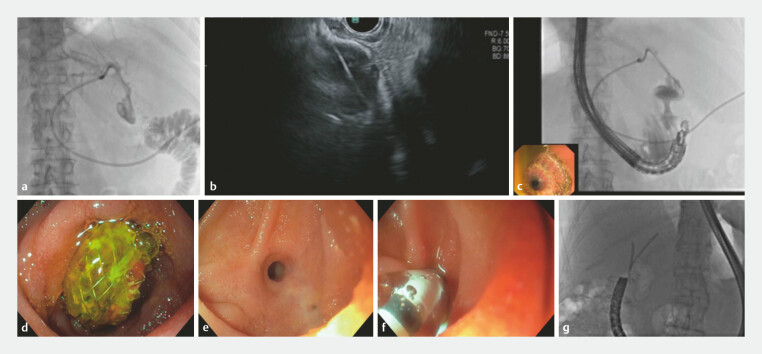
Protocol for EDEE. **a** Filling the target loop via PTBD catheter. **b** EUS-guided anastomosis creation using LAMS. **c** Confirmation of successful LAMS placement. **d** Endoluminal view on EA. **e** Stenotic BDA. **f** Balloon dilation of stenotic BDA. **g** Documentation of inserted stents in stenotic BDA.

In patients with a naïve gastrointestinal anatomy and (malignant) GOO, a nasojejunal catheter was advanced endoscopically beyond the site of the obstruction into the small bowel. In patients with surgically altered anatomy, access to the bilio-enteric limb was established by placing a PTBD catheter. This PTBD catheter was positioned using fluoroscopy to reach the intestinal stump adjacent to the biliary anastomosis. Following successful positioning of the catheter, the target intestinal loop was distended by instilling sterile saline mixed with indigo carmine and, occasionally, contrast medium. In cases of blind loop syndrome, additional filling was performed after puncture using a 19G FNA needle ( Fig. 1 **a** ).

Using a therapeutic longitudinal echoendoscope (UCT-180, Olympus Corporation, Tokyo, Japan), the target loop was identified by visible distension of the lumen and clear visualization of the filling catheter within the lumen. After confirming the target site and administering 1 mg of glucagon for intestinal paralysis, direct puncture was performed using an electrocautery-enhanced delivery system (Hot Axios, Boston Scientific, Marlborough, Massachusetts, United States). Typically, an electrosurgical setting of pure cutting current (Autocut 5.0) was used for tissue penetration (ERBE Elektromedizin, Tübingen, Germany). A single-shot antibiotic was administered perioperatively in all cases. In the setting of GOO, a gastroenteric anastomosis was created. Otherwise, the optimal access route to the target loop was determined at endoscopist discretion. A 20 × 10 mm LAMS was deployed to establish the anastomosis. Neither balloon dilation of the stent nor additional fixation methods were applied in any case ( Fig. 1 **b** )

Following confirmation of successful LAMS placement, the nasojejunal or PTBD catheter was removed either in the same session or following the successful ERCP procedure ( Fig. 1 **c** ). ERCP was performed via LAMS at least 5 days after the index procedure to allow for anastomotic maturation. ERCP was conducted using standard duodenoscopes or therapeutic gastroscopes, depending on the trajectory of the biliary access (TJF-190V and GIF-1TH190, Olympus Corporation, Tokyo, Japan) and conventional accessories, depending on the therapeutic indication (e.g., cannulation and sphincterotomy, stricture dilation, stone extraction, tissue sampling) ( Fig. 1 **d-g** ).

Following uneventful creation of the EA, patients were allowed to resume oral intake beginning with a liquid diet on the same day, followed by gradual advancement to solid food on subsequent days, as clinically appropriate. Post-ERCP dietary recommendations were tailored based on the type and extent of intervention performed.

## Results

A total of 24 patients (70.3 ± 12.6 years; 6 female) were included in the study. Due to the size of the cohort, the following statistical analysis is purely descriptive. Of the 24 enrolled patients, eight patients (33.3%) presented with malignant GOO, whereas 16 patients (66.7%) had surgically altered anatomy. Among the latter, eight patients (50%) had undergone surgery for malignant conditions (five pancreatic cancer, one cholangiocellular carcinoma, two gastric cancer) and eight patients (50%) had undergone surgery for benign diseases, specifically complicated cholecystectomy. Surgical procedures included five Whipple procedures and 11 Roux-en-Y anastomoses. In 14 of 16 patients (87.5%) with altered anatomy, previous attempts at DAE-ERCP had failed. In the two remaining cases with altered anatomy, no attempt at DAE-ERCP was made due to either planned cholangioscopy or electrohydraulic lithotripsy, which were not possible through an enteroscopic approach at that time and, therefore, an EDEE was planned primarily ( Table 1 ).

**Table:** **Table 1** Baseline patient and procedure characteristic of patients with EDEE.

Age	70.3 ± 12.6 years
Sex	Female: n = 6
	Male: n = 18
Surgically altered anatomy	n = 16 (66.7%)
Underlying disease for surgically altered anatomy	Benign: n= 8 (50%)
	Malignant: n=8 (50%)
Surgical procedures	Whipple: n=5 (31.3%)
	Roux-en-Y: n=11 (68.7%)
Previous attempts of DAE-ERCP in patients with surgically altered anatomy	n=14/16 (87.5%)
Pre-existing PTBD	n=15/24 (62.5%)
Indication for EDEE	BDA stenosis: n=10/24
	Blind loop syndrome: n=2/24
	Malignant CBD stenosis: n=8/24
	Tissue acquisition for histopathological diagnosis: n=1/24
	Cholelithiasis: n=3/24
BDA, biliodigestive anastomosis; CBD, common bile duct; DAE-ERCP, device-assisted enteroscopy-assisted endoscopic retrograde cholangiopancreatography; PTBD, percutaneous biliary drainage.	

In the group of patients with non‑altered anatomy, five of eight patients (62.5%) underwent EA primarily because of symptomatic GOO, with the indication for biliary intervention arising only at a later stage. In contrast, among patients with surgically altered anatomy, all individuals (16/16; 100%) underwent EA exclusively to facilitate access for subsequent ERCP. A total of 19 gastroenterostomies and five enteroenterostomies were performed for EA. In the cohort with GOO, a gastrojejunostomy was performed in all cases (8/8; 100%). Among patients with surgically altered anatomy, 11 of 16 (68.8%) underwent gastrojejunostomy, four of 16 (25%) duodenojejunostomy, and one of 16 (6.3%) jejunojejunostomy. In all cases, a 20 × 10 mm LAMS was deployed. Identification and filling of the intestinal loop for EA was achieved using PTBD in 15 patients (62.5%), nasoduodenal/-jejunal catheter placement in eight patients (33.3%), and direct EUS-guided puncture in one patient (4.2%). In patients with GOO, the nasoduodenal/-jejunal catheter was used exclusively (8/8; 100%). Primary stent misplacement occurred in one patient (4.2%), but successful EA was achieved during a second session, resulting in a technical success rate for creation of an EA per patient of 100% (24/24).

No LAMS dislocations occurred during subsequent ERCP procedures; however, spontaneous stent migration was observed in three patients (12.5%) during the follow-up period (follow-up in months: mean = 7,6; median = 4,9; range = 0–28,6, mean time between two follow-ups 77.2 days) *.* In two additional cases, the LAMS was intentionally removed: 1) due to the inability to intubate the afferent loop with the stent in place; and 2) because the LAMS migrated into the massive dilated common bile duct. In all five cases in which the LAMS migrated/was removed, the fistula was preserved by inserting two double pigtail stents. During follow-up, four of the five patients (80%) developed progressive narrowing of the fistula. This prompted need for fistula dilation during subsequent interventions, which was performed in seven sessions overall, corresponding to 9.3% of the 75 follow-up procedures in the full cohort. In six of these seven sessions, dilation was performed directly without prior stenting of the fistula, whereas in one case, a previously placed LAMS was used for fistula stabilization prior to dilation. During two of the seven dilations – both performed without prior fistula stenting – a deep dehiscence occurred (28.6%), necessitating placement of a new LAMS. Overall, fistula dilation during follow‑up resulted in deep dehiscence in two of four affected patients (50%) ( Table 2 ).

**Table:** **Table 2** LAMS migration, removal, and fistula outcomes.

**Parameter**	**Value**
LAMS dislocation during ERCP	n = 0/24 (0%)
Spontaneous LAMS migration	n = 3/24 (12.5%)
Intentional LAMS removal	n = 2/24 (8.3%)
Fistula preservation method	Two double pigtail stents in all 5 cases
Dilation sessions performed during follow-up	n = 7/75 (9.3%)
Dilation technique	Direct dilation without prior stenting: n = 6/7 (85.7%)
	Dilation with prior stabilization by placing a LAMS: n = 1/7 (14.3%)
Deep dehiscence after direct dilation	n = 2/6 (33.3%)
Deep dehiscence after dilation with prior stabilization	n = 0/1 (0%)
ERCP, endoscopic retrograde cholangiopancreatography; LAMS, lumen apposing metal stent.	

All patients underwent EDEE in a staged approach, with a median interval of 29.5 days (range 5–143) between EA and ERCP. Indications for ERCP included biliodigestive anastomotic stenosis (ten patients), blind loop syndrome (two patients), malignant common hepatic duct stenosis (eight patients), tissue acquisition for histopathological diagnosis (one patient) and cholelithiasis (three patients). During ERCP, balloon dilation was performed in 13 patients, cholangioscopy in two, brush sampling in one, and balloon retrieval of stones and sludge in six patients. Stents were placed in 21 patients (n = 5 fully covered SEMS; n = 16 plastic stents). ERCP ultimately failed in three of 24 cases (12.5%) due to inaccessibility of the papilla or BDA. Accordingly, because creation of the EA was successful in all patients and ERCP via LAMS in 21 of 24 patients (87.5%), the overall technical success rate for EDEE was 87.5% (21/24).

Clinical success was achieved in all cases in which EDEE was technically successful. The PTBD could be removed in all cases following successful EDEE. An EDEE procedure-related complication occurred in two patient (8.3%). One patient developed aspiration pneumonia, which was successfully treated with antibiotics. In the other patient, primary LAMS misplacement occurred, but EA was successfully created in a second session ( Table 3 ).

**Table:** **Table 3** Technical and clinical outcomes of EDEE procedures.

Successful EA	100% (24/24)
Successful ERCP via LAMS	87.5% (21/24)
Overall technical success (successful creation of EA followed by successful ERCP via LAMS)	87.5% (21/24)
Clinical success	87.5% (21/24)
Complication rate	8.3% (2/24)
EA, endoscopic ultrasound-guided anastomosis; EDEE, endoscopic ultrasound-directed transenteric endoscopic retrograde cholangiopancreatography; ERCP; ERCP, endoscopic retrograde cholangiography; LAMS, lumen apposing metal stent.	

## Discussion

In patients with surgically altered anatomy, the optimal approach for ERCP remains controversial. Data show that DAE-ERCP and PTBD are comparably successful (87.3% vs. 87.3%), but PTBD carries a higher complication rate (5.7% vs. 29%). Laparoscopy-assisted ERCP achieves higher technical success (99.1%) but at the cost of increased adverse events (AEs) compared with DAE-ERCP (15.1% vs. 5.7%) 27 28 . EUS-directed techniques for biliary interventions with an inaccessible biliary system in (malignant) GOO or altered anatomy usually target the biliary tree directly (EUS-HGA, EUS-CDS). A novel approach using LAMS creates a transmural shortcut, which brings the target site (papilla or BDA) back into reach of conventional endoscopy and allows for standard ERCP techniques including cholangioscopy and recurrent interventions, e.g. for multistenting calibration of strictures. Although EDGE-procedures targeting the large remnant stomach are usually technically less challenging and today are considered a first-line approach for patients requiring biliary interventions following RYGB 29 , available data for EDEE, whicih targets the small intestine, are limited.

To evaluate efficacy and safety of EDEE, it is essential to define the group of patients for whom this approach is beneficial. Although earlier studies primarily included patients with surgically altered anatomy, this analysis demonstrates that EDEE can also be successfully applied in patients with naïve biliary anatomy complicated by (malignant) GOO. Biliary drainage in malignant GOO is usually achieved by creating a double bypass with an additional EUS-HGA following EA. Using the EDEE technique, conventional transpapillary drainage can be performed without need for more invasive transmural access. This expands potential applications of EDEE beyond those of prior cohorts. Moreover, the underlying disease (benign versus malignant) should be considered when assessing eligibility for EDEE. In contrast to the study by Mutignani et al. 25 , which excluded patients with malignant biliary strictures, the current study and those by Ichkhanian et al. 24 and Pérez-Cuadrado-Robles et al. 26 have shown that EDEE can be successfully performed in patients with both benign and malignant pathologies.

A critical prerequisite for EDEE is creation of a safe and effective EA. Accurate identification and distension of the target intestinal loop are crucial for procedure success. In the study by Mutignani et al. 25 , PTBD was the most employed technique for identifying the correct loop. The second most frequently used method involved retrograde placement of a 7F endoscopic tube into the jejunal loop. Additional approaches included transgastric EUS-guided puncture of the left hepatic duct and direct EUS-guided puncture of the jejunal loop. In this study, PTBD and placement of a nasoduodenal/-jejunal tube were the most frequently used methods, with direct EUS-guided puncture reserved for one case involving blind loop syndrome. These findings suggest that a range of effective strategies exist for loop identification and filling, and that the choice should be guided by patient-specific anatomical factors, prior interventions, and endoscopist experience. In terms of technical performance, EA was successfully performed in all patients (100%) in this cohort. This mirrors the high success rates reported in other studies, such as those by Mutignani et al. (100%) 25 , Ichkhanian et al. (98.8%) 24 , and Pérez-Cuadrado-Robles et al. 26 , underscoring that EA is a reliable and reproducible technique, particularly in experienced, high-volume centers.

Once the LAMS is correctly positioned, one of the key concerns is risk of stent migration. According to the European Society of Gastrointestinal Endoscopy guidelines referring to EDGE, delaying EDGE for at least 7 days following LAMS placement, whenever possible, is recommended to reduce likelihood of migration 30 . In urgent situations in which early EDGE was necessary, various techniques to secure the LAMS have been evaluated in studies, including over-the-scope clipping and endoscopic suturing 31 32 . Although the studies mentioned refer to the EDGE procedure, these results may be transferred to the EDEE procedure, because an anastomosis is created first and subsequently ERCP is performed via this anastomosis. In this study, no stent fixation techniques were employed because all procedures followed a two-step approach with separate sessions for EA and ERCP. The shortest interval between the two interventions was 5 days. Importantly, no cases of LAMS migration were associated with the subsequent ERCP procedure itself. This observation is consistent with findings from recent state-of-the art reviews on EUS -directed interventions, which suggests that delaying subsequent interventions by approximately 1 to 2 weeks allows for adequate fistula maturation and thereby reduces risk of spontaneous LAMS migration 33 .

However, spontaneous stent migration was observed in three patients in this study. The strategy of preserving the fistula by placing two double pigtail stents across the anastomosis should be critically evaluated because we found this approach to be associated with a tendency toward developing anastomotic stenosis. Notably, dilation of the stenotic fistula carries a 28.6% risk of deep dehiscence. In conclusion, the available data may suggest that maintaining the LAMS in place for the duration of planned ERCP sessions may be beneficial. Fixation of the LAMS could represent a feasible strategy to reduce risk of migration. If migration does occur and dilation of a stenotic anastomosis is required, placement of a new LAMS prior to dilation may help minimize risk of deep dehiscence. However, these considerations should be interpreted with caution because they are based on retrospective observations and require confirmation in prospective studies.

Subsequent ERCP was technically successful in 21 of 24 patients (87.5%) in this study. Comparable studies have reported EDEE technical success rates ranging from 87.3% 26 to 100% 24 25 , emphasizing that EDEE is an effective technique. Failures of EDEE in this study were due to inability to reach the papilla or BDA endoscopically. In two of these three cases, initial LAMS placement was indicated for resolution of a malignant GOO. These outcomes suggest that even when the primary indication for gastroenteric anastomosis is duodenal obstruction, the site of LAMS placement should ideally be oriented toward the ligament of Treitz, allowing for easier and straight-forward access to the excluded duodenum and the papilla. This forward-looking approach could ensure endoscopic access for potential future biliary interventions.

Patients with complex and multiple biliary strictures represent a challenging cohort for biliary interventions. PTBD has notable limitations in these cases, especially the association with repeated interventions over extended periods and the potential for long-term external drainage, which negatively impacts patient quality of life (QoL) 34 . Similarly, EUS-guided biliary drainage can be suboptimal in cases with multiple strictures at the level of the BDA or when the right biliary system is involved. The key advantage of EDEE lies in the ability to use standard scopes and the full array of endoscopic devices. This facilitates access to both the left and the right biliary systems and enables comprehensive interventions, such as balloon dilation, tissue sampling, cholangioscopy, and complex stenting strategies. As demonstrated in this study, EDEE allows for versatile and effective management of intricate biliary anatomies.

However, the most important outcome is clinical success for the patient. In this cohort, in all patients who underwent successful EDEE, a preexisting PTBD was successfully removed, highlighting a substantial benefit to patient QoL. Although QoL assessment was not an endpoint of this study, which reinforces application of EDEE as a means not only to achieve biliary access but also to eliminate need for more invasive and burdensome drainage methods.

An analysis of procedure safety in this cohort demonstrated no major complications and a low overall complication rate of 8.3% (2/24 patients), with one case of primary stent misplacement and one case of aspiration pneumonia. Patients with malignant GOO, in particular, are at higher risk for aspiration. This underscores the importance of carefully selecting the method of sedation – whether deep sedation or general anesthesia – and considering preprocedure gastric decompression to minimize aspiration risk.

Compared with other studies, Pérez-Cuadrado-Robles et al. 26 reported a higher overall complication rate of 20%. Hereby LAMS-related complications could all be managed conservatively or endoscopically because post-ERCP complications typical of potential risks, such as post-ERCP pancreatitis, post-ERCP cholangitis and post-sphincterotomy bleeding, occurred. Similar to our findings, Mutignani et al. 25 reported no major AEs, and Ichkhanian et al. 24 observed only minimal complications. Overall, these results suggest that EDEE, when performed in experienced centers, has a favorable safety profile with a low risk of severe complications.

Results of the present study should be interpreted with precaution and cannot not be generalized to common practice because of the retrospective design, enrolling patients only from a single, tertiary referral endoscopic expert center. However, to our knowledge, our cohort represents the largest published single-center cohort, evaluating the novel endoscopic approach in a variety of conditions within a real-world scenario also highlighting the benefit of a standardized technical approach to ensure technical and ultimately clinical success.

## Conclusions

EDEE appears to be a technically feasible and clinically effective option for achieving biliary access in carefully selected patients with malignant GOO or surgically altered anatomy. The favorable technical and clinical outcomes observed in this cohort suggest that EDEE may have a role in highly specialized settings when conventional or DAE‑ERCP are not feasible. However, given the retrospective design, limited sample size, and substantial expertise required to perform these procedures safely, these findings should be interpreted cautiously. EDEE should be considered an advanced technique reserved for expert centers rather than a broadly applicable alternative to established ERCP approaches.
